# A Brief History of Bunyaviral Family *Hantaviridae*

**DOI:** 10.3390/diseases11010038

**Published:** 2023-02-28

**Authors:** Jens H. Kuhn, Connie S. Schmaljohn

**Affiliations:** Integrated Research Facility at Fort Detrick, Division of Clinical Research, National Institute of Allergy and Infectious Diseases, National Institutes of Health, Fort Detrick, Frederick, MD 21702, USA

**Keywords:** actinovirus, agnathovirus, hantavirus, hantavirus pulmonary syndrome, loanvirus, mobatvirus, hemorrhagic fever with renal syndrome, orthohantavirus, reptillovirus, thottimvirus

## Abstract

The discovery of Hantaan virus as an etiologic agent of hemorrhagic fever with renal syndrome in South Korea in 1978 led to identification of related pathogenic and nonpathogenic rodent-borne viruses in Asia and Europe. Their global distribution was recognized in 1993 after connecting newly discovered relatives of these viruses to hantavirus pulmonary syndrome in the Americas. The 1971 description of the shrew-infecting Hantaan-virus-like Thottapalayam virus was long considered an anomaly. Today, this virus and many others that infect eulipotyphlans, bats, fish, rodents, and reptiles are classified among several genera in the continuously expanding family *Hantaviridae*.

## 1. Introduction

What is a “hantavirus”? In 1983, the answer seemed relatively straightforward: Less than a dozen serologically, morphologically, and genetically related non-arthropod-borne viruses, each associated with distinct persistently infected rodent hosts, constituted a tight virus clade related to, but separate from, similar viruses carried by or transmitted by arthropod vectors. These rodent-borne viruses were known to be transmitted among rodents and occasionally to humans in aerosols of rodent urine, feces, or saliva or through biting/wounding. Several hantaviruses were found to cause a mild, moderate, or severe disease in humans called “hemorrhagic fever with renal syndrome (HFRS)”, with humans being dead-end hosts [1,2,3]. Accordingly, a new genus was proposed for this group of viruses and named *Hantavirus* after the founding member, Hantaan virus (HTNV) [3,4]. This genus was accepted by the International Committee on Taxonomy of Viruses (ICTV) in 1987 and included in the family *Bunyaviridae* [5,6].

In 1993, a novel hantavirus was associated with a highly lethal acute respiratory distress syndrome, hantavirus pulmonary syndrome, in the southwestern U.S., spurring intense additional hantavirus research and rapid progress in the identification of many more hantaviruses around the world [7,8]. Each novel discovery in relation to these viruses eroded previous definitions of the term “hantavirus”. Here, we briefly recount the story of these viruses, from the first isolation of HTNV to the promotion of the genus *Hantavirus* in the family *Bunyaviridae* to the family *Hantaviridae* in the order *Bunyavirales* [9]. This is not intended to be a comprehensive review of *Hantaviridae* but rather a primer on the history and possible future of this family of viruses.

## 2. Diseases in Search of Viruses

“Korean hemorrhagic fever”, also called “epidemic hemorrhagic fever”, was a disease of unknown etiology that occurred among several thousand United Nations troops during the Korean War (1950–1953). At the height of the conflict, in 1951, hundreds of U.S. military personnel were hospitalized with fever and oral, nasal, and internal hemorrhages; these cases sometimes led to fatalities related to renal failure and shock [10,11]. There was a long history of cases with similar clinical presentation: The disease was likely first outlined in the Yellow Emperor’s Internal Canon (黃帝內經) in Imperial China during the Warring States Period (475–221 BCE) [12] and then noted during World War I after affecting British soldiers in Flanders, Belgium [13,14,15]. The first definite clinical/scientific descriptions came from Asia and Europe in and after 1932 [16]. However, there was no known relationship among them, nor was any causative pathogen identified for any of these diseases.

An important breakthrough was made in 1976, when antigen in the lungs of striped field mice (*Apodemus agrarius* (Pallas, 1771)), trapped in several locations of South Korea, was shown to react with antibodies in sera from Korean hemorrhagic fever patients. Although an agent could not be isolated in cell culture at that time, seven successive passages of antigen-positive lung tissue in adult striped field mice demonstrated that an infectious agent was present. Antisera from patients with various hemorrhagic fevers of known etiologies did not react with this agent, implying that it was likely novel [17,18,19].

### 2.1. The Beginning of the Hantavirology

Propagation of the novel virus in cell culture was finally reported in 1981 [20]. The virus, termed “KHF strain 76-118”, originated from naturally infected rodents trapped near Songnaeri (송내리) and passed through naïve striped field mice four times prior to the isolation attempt. The isolated virus was renamed “Hantaan virus, strain 76-118” after the Hantan River, (한탄강), a tributary of the Imjin River (임진강), which crosses the Demilitarized Zone that separates North Korea and South Korea.

The initial clue to the taxonomic position of HTNV came from electron microscopic examination of HTNV particles, which revealed a morphology akin to that of particles of other viruses then classified in the family *Bunyaviridae* [21,22]. This presumptive evidence was soon corroborated by the biochemical characterization of purified HTNV particles and viral components. Consistent with other viruses in that family, HTNV particles were enveloped and contained three separate nucleocapsid structures encapsulating distinct genomic RNAs [2]. These findings were surprising, as most scientists studying Korean/epidemic hemorrhagic fever expected the etiologic agent to be related to viruses causing Argentinian or Bolivian hemorrhagic fevers (i.e., Junín virus and Machupo virus, respectively, of the family *Arenaviridae*). This expectation was rooted in findings that HTNV, similar to Junín and Machupo viruses, is carried and transmitted by rodents, whereas all then-known members of the family *Bunyaviridae* were vectored by insects or ticks.

Around the time of the initial HTNV characterization, it became clear that this virus was the founding member of a larger group. By 1980, studies had confirmed that viruses closely related to or identical to HTNV caused long-known diseases with similar clinical presentations as ‘Korean/epidemic hemorrhagic fever’ in China, Japan, Scandinavia, and the USSR [17,23,24,25,26,27]. In 1982, the World Health Organization (WHO) sponsored a conference to discuss these diseases, which were known by a variety of names (e.g., hemorrhagic nephroso-nephritis and nephropathia epidemica) [28]. A recommendation was made that all of these diseases be collectively re-termed “haemorrhagic fever with renal syndrome (HFRS)” [29], and this term is still the official disease name in today’s 11th revision of WHO’s International Classification of Diseases (ICD-11), under the “hantavirus disease” subcode 1D62.0 [30].

### 2.2. A New Genus

Molecular and antigenic characterization of HTNV revealed additional properties consistent with viruses classified in the *Bunyaviridae* family, such as ribonuclease-sensitive nucleocapsids, a virion-associated polymerase, and two envelope glycoproteins [2,4,31,32]. However, HTNV did not serologically cross-react with viruses from the four then-recognized genera in the *Bunyaviridae* family (*Bunyavirus*, *Nairovirus*, *Phlebovirus*, and *Uukuvirus*), indicating that the epitopes and HTNV are distinct. The viruses assigned to these genera have conserved 3′ and 5′ complementary nucleotides on each of their three genome segments that form panhandle-like secondary structures important for viral transcription and replication. These nucleotides were identical in the genomes of all viruses of a given genus but differed among genera. Indeed, sequencing of the HTNV genome segments revealed a conserved string of 3′ nucleotides among all three segments, but these were distinct from those in the genomes of viruses assigned to the established genera. This finding led to the suggestion in 1983–1984 that HTNV and related viruses should form a new genus [2,3].

By 1985, several other HTNV-like viruses (e.g., those that today are known as Prospect Hill virus [PHV], Puumala virus [PUUV], and Seoul virus [SEOV]) had been isolated from various rodents and clinical samples from HFRS patients. A collaborative effort to characterize these viruses further supported the need for a unique genus [33]. A proposal was submitted to the ICTV, which accepted the addition of the new genus *Hantavirus* to the family *Bunyaviridae* in 1987 [6]. The Fifth ICTV Report of 1991 described the genus as follows:

“There is one recognized group within the genus *Hantavirus* (at least six viruses), plus a large number of isolates not yet assigned to an antigenic complex; serologically unrelated to members of other genera; probably no arthropod vector involved in transmission”

[5]

From then on, the genus *Hantavirus* changed through the addition and sometimes removal of rodent-borne viruses. In line with nomenclature conventions applied to other genera, the members of the genus *Hantavirus* were henceforth referred to as “hantaviruses”.

### 2.3. A New Disease

In 1993, hantavirology took an unexpected turn when the etiology of a highly lethal acute respiratory distress syndrome in humans living in the southwestern U.S. was found to be a hantavirus [7,8,34] distinct from all then-known hantaviruses [8,35,36]. Since the disease occurred in the Four Corners region of the U.S. (where Arizona, Colorado, New Mexico, and Utah intersect), the virus was originally named “Four Corners virus” [36]. This name and several others brought forward by researchers did not last due to various concerns about political implications. Finally, the U.S. Centers for Disease Control and Prevention (CDC) settled on the name “Sin Nombre virus (SNV)”. The new disease (later traced back to at least 1959 [37]) was termed “hantavirus pulmonary syndrome (HPS)” [7], and this name is still in use today (ICD-11 hantavirus disease subcode 1D62.0 [30]). SNV was first identified as being carried by North American deermice (former *Peromyscus maniculatus* (J. A. Wagner, 1845, sensu lato)) [38], it is known to comprise two separate clades: eastern deermice (*Peromyscus maniculatus* (J. A. Wagner, 1845, sensu stricto)) and western deermice (*Peromyscus sonoriensis* (J. A. Wagner, 1845)) [39]. However, recent evidence indicates that deermice of many more species are susceptible to SNV infection [40].

During subsequent years, many additional HPS-causing and presumed apathogenic relatives were identified in rodents of several other species throughout the Americas. Andes virus (ANDV) was identified in 1996 in South America and is carried by long-tailed colilargos (*Oligoryzomys longicaudatus* (Bennett, 1832)). Several closely related, if not identical, viruses stand out as being the only hantaviruses known to be associated with person-to-person transmission [41,42,43,44,45].

## 3. Viruses in Search of Diseases

The original concept that hantaviruses were strictly associated with rodents was challenged in the early 1990s by the realization that Thottapalayam virus (TPMV), isolated in 1965 from a soricid Asian house shrew (*Suncus murinus* (Linnaeus, 1766)) in India [46], was clearly related to the then-known hantaviruses [5,47,48,49]. Long ignored as a possible artifact, the ecological association of TPMV with shrews was confirmed in 2007 [50], thereby adding eulipotyphlans to the hantavirus host spectrum. Evidence of hantaviruses other than TPMV possibly infecting soricid shrews and talpid moles was accumulated between 1983 and 1990 [51,52,53,54,55,56,57,58,59,60]. However, in 2007, the first unequivocal identifications of non-TPMV soricid hantaviruses were reported via the description of Seewis virus (SWSV) in common shrews (*Sorex* (*Sorex*) *araneus* Linnaeus, 1758), captured in Switzerland [61]; “Camp Ripley virus” in northern short-tailed shrews (*Blarina brevicauda* (Say, 1823)), captured in the U.S. [62]; Cao Bằng virus (CBNV) in Chinese mole shrews (*Anourosorex squamipes* Milne-Edwards, 1872), captured in Vietnam [63]; and “Tanganya virus” in the Therese’s shrews (*Crocidura theresae* Heim de Balsac, 1968), captured in Guinea [64]. Since then, at least another 13 soricid hantaviruses have been discovered in Africa, Asia, Europe, and North America [65]. In 2008, the first talpid hantavirus, Asama virus (ASAV), was described after its discovery in Japanese shrew moles (*Urotrichus talpoides* Temminck, 1841) [66]. At least seven additional talpid mole viruses have since been discovered in Asia, Europe, and North America [65,67,68].

Hantaviruses have been suspected of infecting bats (order Chiroptera) in addition to mammals of the orders Rodentia and Eulipotyphla since 1994 [69,70]. The first confirmation of this hypothesis was published in 2012, when “Magboi virus” was discovered in nycterid hairy slit-faced bats (*Nycteris hispida* (Schreber, 1775)), sampled in Sierra Leone [71]. At least 11 other bat hantaviruses were discovered thereafter in Africa, Asia, and Europe, spanning hosts of both chiropteran suborders Yinpterochiroptera (Hipposideridae, Pteropodidae, and Rhinolophidae) and Yangchiroptera (Emballonuridae, Molossidae, Nycteridae, and Vespertilionidae) [65,72,73].

Hantavirology took another unexpected turn with the metagenomic discovery of three distinct hantaviruses in saltwater actinopterygiid fish of three orders, captured in the South China Sea; one new hantavirus in freshwater actinopterygiid fish, sampled in Europe; one new hantavirus in myxinid fish, captured in the South China Sea; and one new hantavirus in a gekkotan reptile [74,75]. The most recent discovery of additional hantavirus nucleic acids in Australian actinopterygiid freshwater fish and scincomorphan reptiles [76,77,78] suggests that there are many more fish and reptile hantaviruses.

Together, these findings finally abolished the “rodent virus” and “mammal virus” monikers for hantaviruses. At least for now, one dogma still stands: Only rodent-borne hantaviruses have been associated with human disease. Whether the other hantaviruses also have the potential for human spillover or whether they cause diseases in animals other than humans remains to be determined through careful study of these mostly completely uncharacterized agents.

## 4. A New Taxonomy

The hantavirus clade was not the only virus group that greatly expanded over recent decades. Hundreds of novel viruses were easily assignable to the *Bunyaviridae* family, and dozens of them could be included in the four established *Hantavirus* sister genera (*Bunyavirus*, *Nairovirus*, *Phlebovirus* [by then including the members of then-abolished genus *Uukuvirus*], and *Tospovirus*). With this expansion, the advent of improved genome sequencing methodologies, and new tools to probe phylogeny, came the realization that the true diversity and the evolutionary relationship of all these viruses could not be adequately depicted in a three-taxon (family, genus, species) hierarchy; distinct subclades of viruses became discernible in each of the genera, and entire, complex, new virus clades, apparently sister to the established genera, needed to be added. In 2017, family *Bunyaviridae* was therefore promoted to the *Bunyavirales* order; in parallel, the five genera were promoted to families (*Bunyavirus*→*Peribunyaviridae*; *Hantavirus*→*Hantaviridae*; *Nairovirus*→*Nairoviridae*; *Phlebovirus*→*Phenuiviridae*; and *Tospovirus*→*Tospoviridae*) to enable high-resolution classification of their members at the genus rank [9].

To remove possible ambiguities associated with the vernacular term “hantavirus”, a new genus, *Orthohantavirus*, was created within *Hantaviridae* in 2018 [9]. Thereafter, genus members were to be referred to as “orthohantaviruses” and family members as “hantaviruses”, or, preferably, “hantavirids”, using the vernacular family-specific suffix “-virid(s)” to clarify family references in communication [79]. Importantly, throughout the dramatic expansion of known bunyavirals, hantavirids remained a highly distinct and therefore easily identifiable clade, and DivErsity pArtitioning by hieRarchical Clustering (DEmARC) analysis confirmed most well-characterized hantavirids to be clearly distinct entities [80]. Ironically, this analysis also revealed that eulipotyphlan TPMV formed a sister group with most other eulipotyphlan and all rodent orthohantaviruses. Bat viruses fell into separate sister clades. Thus, in 2019, the family was expanded by three genera: *Loanvirus* (bat viruses), *Mobatvirus* (bat and eulipotyphlan viruses), and *Thottimvirus* (TPMV and other eulipotyphlan relatives) [81]. Furthermore, in 2019, genera *Actinovirus* (Actinopterygiid viruses), *Agnathovirus* (Myxinid viruses), and *Reptillovirus* (Reptilian viruses) were added to the family, which was further subdivided into four subfamilies: *Acanthavirinae* (*Actinovirus*), *Agantavirinae* (*Agnathovirus*), *Mammantavirinae* (*Loanvirus*, *Mobatvirus*, *Orthohantavirus*, *Thottimvirus*), and *Repantavirinae* (*Reptillovirus*) [82]. The most up-to-date composition of *Hantaviridae* [83] is outlined in Table 1.

**Table 1:** 2022 [83] and projected 2023 [84] taxonomies of the bunyaviral family *Hantaviridae*.

Genus	Species Name (2022)	Projected Species Name (2023)	Virus Name (Abbreviation)	Human Disease
**Subfamily *Acanthavirinae* (hosted by fish)**				**/**
*Actinovirus*	*Batfish actinovirus*	*Actinovirus halieutaeae*	Wēnlǐng minipizza batfish virus (WEMBV)	**/**
	*Goosefish actinovirus*	*Actinovirus lophii*	Wēnlǐng yellow goosefish virus (WEYGV)	**/**
	*Perch actinovirus*	*Actinovirus bernense*	Bern perch virus (BRPV)	**/**
	*Spikefish actinovirus*	*Actinovirus triacanthodis*	Wēnlǐng red spikefish virus (WERSV)	**/**
**Subfamily *Agantavirinae* (hosted by fish)**				
*Agnathovirus*	*Hagfish agnathovirus*	*Agnathovirus eptatreti*	Wēnlǐng hagfish virus (WEHV)	**/**
**Subfamily *Mammantavirinae* (hosted by bats, moles, shrews, and rodents)**				
*Loanvirus*	*Brno loanvirus*	*Loanvirus brunaense*	Brno virus (BRNV)	/
	*Longquan loanvirus*	*Loanvirus longquanense*	Lóngquán virus (LQUV)	/
*Mobatvirus*	*Laibin mobatvirus*	*Mobatvirus laibinense*	Láibīn virus (LAIV)	/
	*Lena mobatvirus*	*Mobatvirus lenaense*	Lena virus (LENV)	/
	*Nova mobatvirus*	*Mobatvirus novaense*	Nova virus (NVAV)	/
	*Quezon mobatvirus*	*Mobatvirus quezonense*	Quezon virus (QZNV)	/
	*Xuan Son mobatvirus*	*Mobatvirus xuansonense*	Xuân Sơn virus (XSV)	/
*Orthohantavirus*	*Andes orthohantavirus*	*Orthohantavirus andesense*	Andes virus (ANDV)	HPS
			Castelo dos Sonhos virus (CASV)	HPS
			Lechiguanas virus (LECV = LECHV)	HPS
			Orán virus (ORNV)	HPS
	*Asama orthohantavirus*	*Orthohantavirus asamaense*	Asama virus (ASAV)	/
	*Asikkala orthohantavirus*	*Orthohantavirus asikkalaense*	Asikkala virus (ASIV)	/
	*Bayou orthohantavirus*	*Orthohantavirus bayoui*	bayou virus (BAYV)	HPS
			Catacamas virus (CATV)	HPS
	*Black Creek Canal orthohantavirus*	*Orthohantavirus nigrorivense*	Black Creek Canal virus (BCCV)	HPS
	*Bowe orthohantavirus*	*Orthohantavirus boweense*	Bowé virus (BOWV)	/
	*Bruges orthohantavirus*	*Orthohantavirus brugesense*	Bruges virus (BRGV)	/
	*Cano Delgadito orthohantavirus*	*Orthohantavirus delgaditoense*	Caño Delgadito virus (CADV)	/
	*Cao Bang orthohantavirus*	*Orthohantavirus caobangense*	Cao Bằng virus (CBNV)	/
			Liánghé virus (LHEV)	/
	*Choclo orthohantavirus*	*Orthohantavirus chocloense*	Choclo virus (CHOV)	HPS
	*Dabieshan orthohantavirus*	*Orthohantavirus dabieshanense*	Dàbiéshān virus (DBSV)	/
	*Dobrava-Belgrade orthohantavirus*	*Orthohantavirus dobravaense*	Dobrava virus (DOBV)	HFRS
			Kurkino virus (KURV)	HFRS
			Saaremaa virus (SAAV)	HFRS
			Sochi virus (SOCV)	HFRS
	*El Moro Canyon orthohantavirus*	*Orthohantavirus moroense*	Carrizal virus (CARV)	/
			El Moro Canyon virus (ELMCV)	HPS
			Huitzilac virus (HUIV)	/
	*Fugong orthohantavirus*	*Orthohantavirus fugongense*	Fúgòng virus (FUGV)	/
	*Fusong orthohantavirus*	*Orthohantavirus fusongense*	Fǔsōng virus (FUSV)	/
	*Hantaan orthohantavirus*	*Orthohantavirus hantanense*	Amur virus (AMRV)	HFRS
			Hantaan virus (HTNV)	HFRS
			Soochong virus (SOOV)	/
	*Jeju orthohantavirus*	*Orthohantavirus jejuense*	Jeju virus (JJUV)	/
	*Kenkeme orthohantavirus*	*Orthohantavirus kenkemeense*	Kenkeme virus (KKMV)	/
	*Khabarovsk orthohantavirus*	*Orthohantavirus khabarovskense*	Khabarovsk virus (KHAV)	/
			Topografov virus (TOPV)	/
	*Laguna Negra orthohantavirus*	*Orthohantavirus negraense*	Laguna Negra virus (LANV)	HPS
			Maripa virus (MARV)	HPS
			Rio Mamoré virus (RIOMV)	HPS
	*Luxi orthohantavirus*	*Orthohantavirus luxiense*	Lúxī virus (LUXV)	/
	*Maporal orthohantavirus*	*Orthohantavirus maporalense*	Maporal virus (MAPV)	/
	*Montano orthohantavirus*	*Orthohantavirus montanoense*	Montaño virus (MTNV)	/
	*Necocli orthohantavirus*	*Orthohantavirus necocliense*	Necoclí virus (NECV)	/
	*Oxbow orthohantavirus*	*Orthohantavirus oxbowense*	Oxbow virus (OXBV)	/
	*Prospect Hill orthohantavirus*	*Orthohantavirus prospectense*	Prospect Hill virus (PHV)	/
	*Puumala orthohantavirus*	*Orthohantavirus puumalaense*	Hokkaido virus (HOKV)	/
			Muju virus (MUJV)	HFRS
			Puumala virus (PUUV)	HFRS
	*Robina orthohantavirus*	*Orthohantavirus robinaense*	Robina virus (ROBV) *	/
	*Rockport orthohantavirus*	*Orthohantavirus rockportense*	Rockport virus (RKPV)	/
	*Sangassou orthohantavirus*	*Orthohantavirus sangassouense*	Sangassou virus (SANGV)	/
	*Seewis orthohantavirus*	*Orthohantavirus seewisense*	Seewis virus (SWSV)	/
	*Seoul orthohantavirus*	*Orthohantavirus seoulense*	gōu virus (GOUV)	HFRS
			Seoul virus (SEOV)	HFRS
	*Sin Nombre orthohantavirus*	*Orthohantavirus sinnombreense*	New York virus (NYV)	HPS
			Sin Nombre virus (SNV)	HPS
	*Tatenale orthohantavirus*	*Orthohantavirus tatenalense*	Tatenale virus (TATV)	/
	*Thailand orthohantavirus*	*Orthohantavirus thailandense*	Anjozorobe virus (ANJZV)	/
			Serang virus (SERV)	/
			Thailand virus (THAIV)	/
	*Tigray orthohantavirus*	*Orthohantavirus tigrayense*	Tigray virus (TIGV)	/
	*Tula orthohantavirus*	*Orthohantavirus tulaense*	Adler virus (ADLV)	/
			Tula virus (TULV)	HFRS
	*Yakeshi orthohantavirus*	*Orthohantavirus yakeshiense*	Yákèshí virus (YKSV)	/
*Thottimvirus*	*Imjin thottimvirus*	*Thottimvirus imjinense*	Imjin virus (MJNV)	/
	*Thottapalayam thottimvirus*	*Thottimvirus thottapalayamense*	Thottapalayam virus (TPMV)	/
**Subfamily *Repantavirinae* (hosted by reptiles)**				
*Reptillovirus*	*Gecko reptillovirus*	*Reptillovirus hemidactyli*	Hǎinán oriental leaf-toed gecko virus (HOLGV)	/

HFRS, hemorrhagic fever with renal syndrome (ICD-11 hantavirus disease subcode 1D62.0); HPS, hantavirus pulmonary syndrome (ICD-11 hantavirus disease subcode 1D62.1) [30,85]. Unclassified orthohantaviruses associated with HPS are several ANDV-like viruses (e.g., “Araraquara”, “Araucária”, “Bermejo”, “Juquitiba”, “Maciel”, “Paranoá virus”, “Pergamino”, and “Tunari”), “Anajatuba virus” and the SNV-like viruses (“Blue River” and “Monongahela”) [85]. * Robina virus might be a mobatvirus, possibly requiring reclassification [72].

## 5. Evolution of Hantavirids

After the genus *Hantavirus* was established, the idea that each hantavirus was uniquely adapted to a particular, distinct host and that these viruses co-evolved (co-speciated) with their hosts began to take shape [86,87]. The four best-characterized hantaviruses at the time were observed to persistently infect specific/distinct rodent hosts without causing discernible disease: HTNV was known to be hosted by murid striped field mice [17,18,19], PHV by cricetid meadow voles (*Microtus* (*Mynomes*) *pennsylvanicus* (Ord, 1815)) [53], PUUV by cricetid bank voles (*Clethrionomys glareolus* (Schreber, 1780)) [88], and SEOV by murid brown rats (*Rattus norvegicus* (Berkenhout, 1769)) and roof rats (*Rattus rattus* (Linnaeus, 1758)) [89,90], respectively. This close association of one particular hantavirus with one particular rodent host on largely overlapping virus/rodent phylogenetic trees became a frequently repeated pattern, and ultimately a dogma. However, from the beginning, this dogma was not supported scientifically in absolute terms; for example, as outlined above, SEOV was already known to infect at least *two* specific rodents [89]. The already-mentioned susceptibility of various distinct deermice to SNV infection, recognized only after the taxonomy of deermice had to be revised based on molecular evidence [38,39,40], further emphasizes that the natural virus–host connections are not yet well-defined. Even a further relaxed interpretation of co-evolution (i.e., a dogma loosened to virus–host genus association) was challenged: A serologically distinct hantavirus, Dobrava virus (DOBV), was discovered in yellow-necked field mice (*Apodemus flavicollis* (Melchior, 1834)) [91,92,93], i.e., in a different species of *Apodemus* than that harboring HTNV. To stick to the overall idea, TPMV, the virus discovered in soricid shrews [46], had to be ignored or the shrew host had to be explained as a spillover host from an unidentified “true” rodent host. However, after 2007 [50,61,62,63,64], eulipotyphlan hantaviruses could not be ignored anymore.

As exceptions to the one-mammal-one-hantavirus rule became more frequent than the rule itself [94,95], it became apparent that hantavirid co-speciation with hosts is just one factor that influenced hantavirid evolution. Genomic segment reassortment among hantavirids [96], host spillover, and host-switching [97,98,99,100,101,102] have likely occurred many times and obfuscated the evolutionary history of the family *Hantaviridae*. Various theories on hantavirid evolution have been discussed over recent decades [86,87,103,104,105,106,107,108,109,110,111,112,113]. However, none of them included the recently discovered acanthavirins, agantavirins, and repantavirins, rendering them ineffective for the entire family. Mammantavirins (loanviruses, mobatviruses, orthohantaviruses, and thottimviruses) offer themselves to a cohesive analysis because they, at least according to current knowledge, exclusively infect mammals. Their broad infection of bats, eulipotyphlans, and rodents would require the mammantavirin ancestor to be a virus of an early boreoeutherian, i.e., an animal that existed at the estimated diversification point of placental mammals in the Cenozoic (≈66 My ago). However, this assumption may be challenged soon, as accumulating evidence alludes to the existence of marsupial orthohantaviruses [69]. If virus–host co-evolution/co-speciation is a baseline assumption, then inclusion of acanthavirins, agantavirins, and repantavirins moves the hantavirid ancestor at least to the rise of jawless fish, i.e., around or before the Late Carboniferous (≈359–299 My ago). However, this hypothesis brings up the challenging question of why hantavirids were *only* found in a few animal clades rather than in all of them, assuming that they have not simply been massively undersampled.

An alternative hypothesis is that the hantavirid phylogeny is the result of a more recent history, possibly resulting from preferential host-switching and local adaptation rather than parallel host and virus evolution. Evidence supporting this hypothesis includes the finding of specific hantavirids in more than one specific host and the absence of hantavirids in many hosts. Of course, this hypothesis would not explain the origin of mammalian hantavirids, but the origin of bunyavirals [114] suggests ecdysozoans (e.g., arthropods such as insects or arachnids) as early or even current hantavirid hosts. Fitting the latter (heretical) hypothesis are the recent reports of hantavirid-related RNA-directed RNA polymerase-encoding nucleic acids, found by metagenomic sequencing in curculionid diaprepes root weevils (“coleopteran hanta-related virus OKIAV221”) and a perlid stonefly (“plecopteran hanta-related virus OKIAV215”) [115].

## 6. Discussion and Future Directions

So, what is a hantavirid? For now, the answer has become rather unsatisfactory: Hantavirids form a distinct clade of animal-infecting cluster 1 bunyavirals that are closely related to viruses assigned to families *Arenaviridae*, *Cruliviridae*, *Fimoviridae*, *Phasmaviridae*, *Peribunyaviridae*, and *Tospoviridae* [116]. Their evolutionary history is unclear and will likely not be resolved until targeted studies increase confidence in the overall spectrum of hantavirid hosts. The thus-far sporadic discoveries of hantavirids in highly diverse (actinpterygiid and myxinid) fish and (gekkotan and scincomorphan) reptiles [74,75,76,77,78] indicate enormous diversity and imply that hantavirids infect amphibians and possibly crocodilians and their closest relatives, i.e., birds. The latter possibility is supported by at least one publication [117]. The history of the long-ignored soricid TPMV is a reminder that such anecdotal evidence should not be discounted until disproven.

While these larger questions are explored, hantavirologists will also have to increase the resolution of established taxonomies. The genomes of numerous hantavirids (several of which are shown in this review in quotation marks) and the genomes of dozens of “classic” rodent orthohantaviruses have not been completely sequenced. This lack of data not only prevents their official classification [80] but also prevents honing in on such pressing issues as understanding the role of genomic segment reassortment in hantavirid evolution or defining species and species complexes in hantavirid taxonomy. On the host side, recent developments indicate that even the taxonomy of rodents is much less certain than previously thought. With entire genera, such as *Peromyscus*, being reorganized based on molecular evidence [39], the true hantavirid–host relationships will likely have to be re-evaluated entirely. It is possible that these efforts will allow the reestablishment of some prior dogmas, such as the one-hantavirid-one-host hypothesis or the notion that members of individual hantavirid subfamilies only infect particular animal groups (fish, reptiles, or mammals). Alternatively, re-evaluation may require abandonment of any of these as obsolete. Today, only one dogma of yore still stands: All hantaviruses known to cause disease in humans are rodent-borne.

## Data Availability

Not applicable.
